# The Value of Non-Referential Gestures: A Systematic Review of Their Cognitive and Linguistic Effects in Children’s Language Development

**DOI:** 10.3390/children8020148

**Published:** 2021-02-17

**Authors:** Ingrid Vilà-Giménez, Pilar Prieto

**Affiliations:** 1 Department of Translation and Language Sciences, Universitat Pompeu Fabra, 08018 Barcelona, Spain; pilar.prieto@upf.edu; 2 Department of Subject-Specific Education, Universitat de Girona, 17004 Girona, Spain; 3 Institució Catalana de Recerca i Estudis Avançats (ICREA), 08010 Barcelona, Spain

**Keywords:** non-referential gestures, prosody, pragmatics, children, language development, cognitive development, narrative development, information recall, narrative discourse comprehension, oral narrative discourse performance

## Abstract

Speakers produce both referential gestures, which depict properties of a referent, and non-referential gestures, which lack semantic content. While a large number of studies have demonstrated the cognitive and linguistic benefits of referential gestures as well as their precursor and predictive role in both typically developing (TD) and non-TD children, less is known about non-referential gestures in cognitive and complex linguistic domains, such as narrative development. This paper is a systematic review and narrative synthesis of the research concerned with assessing the effects of non-referential gestures in such domains. A search of the literature turned up 11 studies, collectively involving 898 2- to 8-year-old TD children. Although they yielded contradictory evidence, pointing to the need for further investigations, the results of the six studies–in which experimental tasks and materials were pragmatically based–revealed that non-referential gestures not only enhance information recall and narrative comprehension but also act as predictors and causal mechanisms for narrative performance. This suggests that their bootstrapping role in language development is due to the fact that they have important discourse–pragmatic functions that help frame discourse. These findings should be of particular interest to teachers and future studies could extend their impact to non-TD children.

## 1. Introduction

Gesture is a powerful embodied form of communication. Apart from their rich communicative value, gestures have been shown to act as cognitive bootstrappers, as they can contribute to changes in children’s linguistic knowledge (see [1,2,3], for reviews). Understanding the relative value of different types of co-speech gestures is crucial to unraveling how gestures pave the way for language development. However, the field of language development has tended to focus on the role of referential gestures, such as deictic or iconic gestures, which imagistically represent the properties of a referent and thus bear a close relationship to the semantic content of the speech. The value of non-referential gestures, such as hand movements which typically associate with prosodically prominent positions in speech but do not encode specific semantic content, has been comparatively neglected. It is the small amount of research on the latter––non-referential gestures––that we will systematically review in this article, limiting our focus to typically developing children (henceforth TD), since to our knowledge no studies have been conducted on the role of non-referential gestures in non-TD children. By means of this review, we hope to gain insight into the link between the important discourse–framing properties of non-referential gestures and their potential bootstrapping role in cognitive and language development. An understanding of this relationship will allow us to point to some practical implications for the teaching of TD children, as well as ideas for promoting multimodally-based narrative and pragmatic trainings, and some directions for future research. Future investigations could also extend these findings to non-TD children.

Current research has demonstrated that co-speech gestures are tightly linked to speech production and perception, suggesting that the two modalities are very closely intertwined in creating meaning and make up a well-integrated communicative system (see [4,5,6], and many others). Previous studies focusing on referential gestures have shown that children’s gestures serve as forerunners of future linguistic skills in many populations including not only TD children (e.g., [7,8,9]; see [10] for a review), but also late talking toddlers (e.g., [11]), and children with an Autism Spectrum Disorder (ASD) diagnosis (e.g., [12,13,14]; see [15] for a review). It has also been well established by a variety of studies that referential gestures have a positive effect on adults’ and children’s cognitive and linguistic abilities (see [16] for a meta-analysis review), boosting memory recall, for example, in TD children [17,18,19,20,21].

Typically developing children start producing their first gestures, typically deictic or pointing gestures that identify objects, people, events, or locations, between 9 and 12 months of age and before they produce their first words [22,23,24]. These gestures help children carry out successful dyadic interactions with their parents and caregivers (see [25], for a review on the development of deictic pointing in infancy). Already in the transition between the babbling stage and single-word period, infants start to semantically and temporally coordinate their pointing-speech combinations, and gesture is used to complement or reinforce speech [26,27]. Deictic declarative gestures have proven to be a reliable predictor of language skills not only in TD [7,28,29,30], but also in children with speech and language impairments, such as ASD infants [14] (see also [31] for a meta-analysis review). This type of gesture has also been identified as the most markedly impaired in ASD [32,33,34]. Importantly, the fact that deictic gestures place high social and interactive demands on early interactions is an indication that these gestures constitute a powerful tool for early interventions in ASD programs (see also the positive effects of a pointing gesture intervention in generating larger vocabulary repertoires in TD children by [35]).

At this early stage, TD children also start producing iconic gestures that allow them to represent information about a referent in speech, such as an object, an action, or a space. Early iconic gestures are used to depict actions or attributes associated with objects, such as raising arms to indicate big size or flapping arms to represent a bird flying (see [36]) [22,23,37,38]. At around two years of age, there is a sharp increase in the number of iconic gestures produced (e.g., [39,40,41]), corresponding with the period in which children also show an increased sensitivity to iconicity in gesture comprehension [42,43]. For instance, a study assessing spontaneous gestures performed by 40 TD children observed from 14 to 34 months of age reported a spurt in iconic gesture production at roughly 26 months, with children past this threshold not only using iconic gestures more frequently but also employing them to convey a more varied set of meanings [40]. Moreover, a longitudinal study by [41] also found an increase in the production of iconic gestures between 22 and 26 months of age, which were usually used to convey action meanings not yet conveyed in the first verbs (e.g., “go like this” + move fisted empty hand in circles as if stirring, p. 9). Other studies have reported that TD children benefit from observing referential iconic gestures in complex linguistic processes, such as narrative comprehension [44,45]. There is also evidence that a specific type of iconic gesture (“character-viewpoint” or CVPT gestures, in which the gesturer takes on the role of a character in a story; see [6]) can serve as the precursor [46] and predictor [47] of more complex narrative abilities undergoing development. Concerning non-TD children, while research shows that young children with ASD produce deictic gestures less often than TD children, empirical evidence about the role of other forms of gesture in ASD language development comes from only one study. The study by [14] explored the gesture-language relation in autistic children by tracking gesture type production (deictic, give, iconic, or conventional) and subsequent language outcomes of 18-month-old TD and 30-month-old ASD children (*n* = 23). They found that only deictic gestures predicted vocabulary growth in both TD and ASD children, but such gestures occurred at significantly lower prevalence (70% ASD vs. 96% TD) and frequency (45% ASD vs. 60% TD) rates in the ASD group.

Later on, between 2 and 3 years of age, another type of gesture starts to emerge, the non-referential gesture, often called a beat gesture (McNeill [6] describes this type of gesture as being a rhythmically short and quick “simple flick of the hand or fingers up and down, or back and forth” (p. 15) that lacks referentiality and associates with prosodic prominent positions in speech. For this reason, following a McNeillian classification of gestures, many studies have called such gestures “beat” gestures (i.e., as if marking a beat). More recently, this traditional view of non-referentials has been challenged and a more inclusive definition of non-referential gestures has been adopted that emphasizes their rhythmic, pragmatic and discursive properties. Within this view, “beat gestures” are considered as non-referential gestures that do not only act mainly as rhythmic highlighters, but also contribute clear pragmatic and discursive meanings that help frame oral discourse structure [48,49,50,51].) [52,53]. The study by [53] documented the appearance of beat gestures in French-English bilingual children in the period from 2 years to 3 years 6 months of age, and observed that the children began to produce these gestures once they were able to perform sentence-like or more linguistically complex spoken utterances (in other words, when the mean length of their utterances increased). The literature on the acquisition of these gestures is sparse and has focused on how children gesture with non-referential gestures while they are narrating. Some studies have shown that non-referential gestures start appearing in complex narrative discourses at around 4–5 to 6 years of age [6,54,55] (see also [56] for specifically language-impaired children). A cross-sectional study with French-speaking children aged 6 and 10, and adults by [54] found that, in contrast to the average number of representational (i.e., iconic) gestures, the average number of non-representational gestures (i.e., non-referential gestures) increased with age and that these gestures served both discursive functions (by accompanying connectors, highlighting important linguistic units, or performing anaphoric functions) and framing functions. Similarly, another cross-sectional study by [57] with 5- and 10-year-old French, American, and Italian children demonstrated that the older children tended to produce more non-referential gestures that helped to structure speech or mark cohesion in discourse than the younger ones. Recently, a longitudinal study assessing multimodal narrative development in children 5–6 to 7–9 years of age revealed that, in contrast with referential iconic gestures, the use of non-referential gestures increased noticeably with age, as narrative skills matured [58] (see also [59] for similar results).

As mentioned above, the literature on multimodal language development has tended to focus on the value of referential gestures in paving the way for early language development in children, with less attention being paid to the precursor and predictive role of non-referential gestures as well as their bootstrapping impact on language development. The obvious question is whether non-referential gestures have the same beneficial role in cognitive and linguistic dimensions as referential (i.e., deictic and iconic) gestures. This research gap was already noted a decade ago in a meta-analysis study conducted by [16], who called for further research to examine the nature and impact of non-referential gestures.

In our view, there are strong reasons to believe that non-referential gestures are important in children’s language development. Though the classic McNeillian classification of gestures [6] has highlighted the rhythmic character of non-referential “beat” gestures and their consequent link to prosody, the fact that non-referential gestures have been relatively understudied may be due to the general theoretical claim coming from this view that these gestures lack abstract semantic content. Indeed, many studies have assumed that because these gestures lack referential meaning, their contribution to language learning and development is negligible (e.g., [20,45,60,61,62], and others). However, it is well known that non-referential gestures in adult discourse mark information structure and focused information, as well as new or accessible referents [62,63]. In this way, non-referential gestures can act as multimodal pragmatic cues which highlight important linguistic functions in speech that help frame complex oral discourse in later stages of language development ([6,50,51,64,65,66,67], among others) (e.g., [68]). We thus hypothesize that non-referential gestures are important in the processing and acquisition of more complex language skills such as narratives, as they play an important role in framing discourse.

The present review will systematically assess, evaluate, and compare the available research concerned with the scaffolding role of non-referential gestures in three important areas of child learning and language development, namely information recall, narrative discourse comprehension, and oral narrative discourse performance. With regard to the second and third of these areas, it should be noted that the development of narrative discourse abilities as an oral language skill is an important achievement for children, as it has been typically associated with children’s complex linguistic development and successful school literacy [69,70] (see [71] for a review). Simultaneously, we will seek answers for three main research questions related respectively to the association, predictive, and causal effects of non-referential gestures, as follows.

(1) Association effects. Does observing another speaker’s non-referential gestures enhance information recall and narrative discourse comprehension in TD children?
(2) Predictive effects. Does the frequency of use of non-referential gestures by TD children predict better narrative production skills later in development?
(3) Causal effects. Can training TD children with non-referential gestures bring about an improvement in narrative production scores in a subsequent posttest?

## 2. Methods

The Preferred Reporting Items for Systematic Reviews and Meta-Analyses (PRISMA) statement (e.g., [72,73]) guided the methodology and reporting of this systematic review.

### 2.1. Identification of Studies and Inclusion Criteria

**Figure 1:**
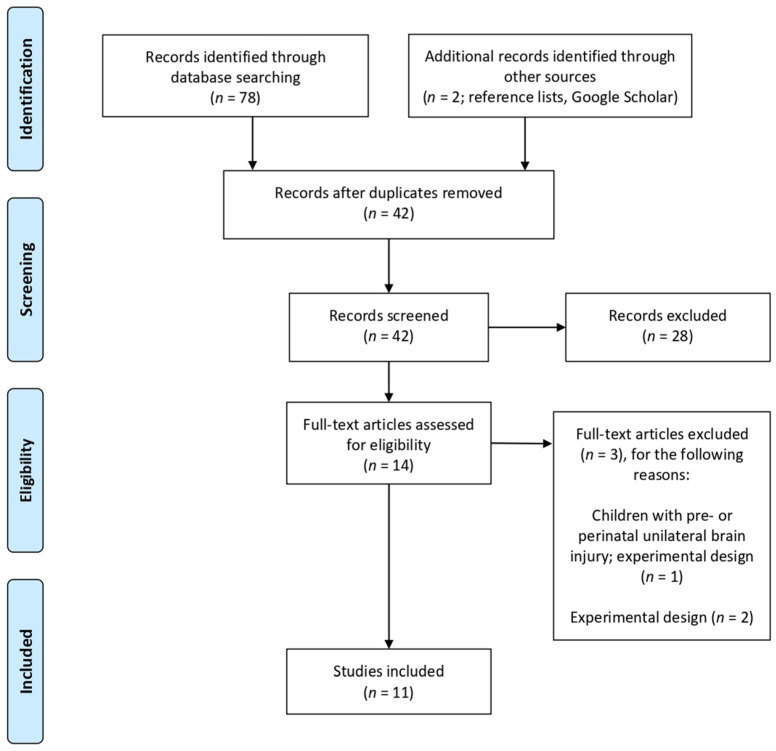
PRISMA Flow Diagram.

As stated above, the principal aim of this systematic review was to address and compare the available research devoted to the effects of non-referential gestures on children’s cognitive and linguistic skills. The process of identifying studies is summarized in the PRISMA flow diagram in Figure 1. First, a comprehensive search strategy was conducted during the second semester of 2020 using four electronic databases: Scopus, Web of Science, PubMed, and PsycInfo. The database searches were limited to English language works dated between 1970 and 2020. Search terms used in the databases included: (“children*” OR “preschooler*”) AND (“beat gesture*” OR “non-referential beat gesture*” OR “non-referential gesture*” OR “non-referential beat*”) AND (“memory recall” OR “recall” OR “comprehension” OR “narrative comprehension” OR “narrative performance” OR “produc*” OR “observ*”). The reference lists of articles retrieved were screened to identify any relevant additional studies on the topic. Both conference proceedings and papers that were under review were also included in the identification process (one conference proceeding paper was identified via Google Scholar).

Once the literature had been identified, the titles and abstracts of the compiled list of retrieved articles were screened for relevance by the first author and duplicated or irrelevant articles were removed using Mendeley reference software. Articles which warranted further examination were selected for full-text review.

To this end, all the potentially relevant articles that were identified and that could answer the questions were assessed as full text independently by the two authors according to the following eligibility criteria, so as to reduce the risk of inclusion bias:

- The article was written in English and published (or under review) in a peer-reviewed journal or in peer-reviewed conference proceedings.
- The study was published by 1970 or later.
- The study reported in the article followed an experimental design yielding quantitative data. Thus, studies which assessed gesture use and development in a merely descriptive fashion without manipulating variables (e.g., [54,55]) were excluded.
- Participants were TD children, aged from 2 to 8 years who did not present any language or developmental disorder that affects communication. To our knowledge, there is no previous research that has dealt with the effects of non-referential gestures in children with language disorders.
- The experimental design involved speech with the presence or absence of non-referential gestures (either by investigating the children’s observation of another speaker’s gestures or the children’s own gesture production) as an isolated variable. Thus, studies that tested the impact of all kinds of gestures simultaneously (i.e., multimodal training studies) were excluded.
- The outcome of the experiment was measured in terms of memory recall, comprehension, or language production.

### 2.2. Data Extraction

The assessment process yielded a total of 11 full text articles which met the inclusion criteria. All were published between 2012 and 2020 except for one, which is currently undergoing the final review process prior to publication. Likewise, in all 11 studies a non-referential gesture experimental condition was compared to other gesture or no gesture conditions. The total sample size of all the studies was 898 children between 2 and 8 years of age whose native language was English, Catalan (Catalan-Spanish bilingual), or Turkish. One study was conducted in Singapore, three in Australia, six in Catalonia, and one in Turkey. The Standard Quality Assessment criteria for evaluating primary research papers from a variety of fields (Kmet checklist Appraisal Tool) was used to assess the methodological quality of the studies [74]. The overall quality of the studies was found to be acceptable, with clearly stated research questions and appropriately used experimental methods.

For each study, the following information was extracted:

- Reference: author(s) and year of publication
- Aim of the study
- Study population, including number, gender, age range (mean and standard deviation), and language of participants
- Study design
- Control and experimental conditions
- Outcome measure
- Main results

This data (except study aims) can be seen in Table 1, with the main results framed in terms of the extent to which they answered the question “Did non-referential gestures have a positive effect on children’s outcome measure?” All the information summarized in the tabular form was observed to find the main similarities between studies meeting the inclusion criteria.

Formal meta-analysis was not considered feasible and therefore not undertaken due to the low number of studies and to the fact that the study designs and reported outcome measures varied markedly. Thus, a narrative synthesis seemed to be the most appropriate way to compare the results of these studies and draw overall conclusions.

## 3. Results

Because these 11 studies did not use the same outcome measures, they were divided into three groups on that basis for purposes of comparison. It is important to mention that only comparable variables within experimental designs were extracted for all studies. Below, we first compare studies that reported results related to the association effects between non-referential gestures and children’s cognitive and linguistic abilities, such as information recall and narrative discourse comprehension (Section 3.1). Next, we compare studies that examined the predictive value of non-referential gestures in children’s later narrative productions (Section 3.2). Finally, we compare studies that examined the causal effects of gesture training paradigms using non-referential gestures on children’s oral narrative discourse performance (Section 3.3).

### 3.1. Association Effects

#### 3.1.1. Information Recall

Seven of the 11 studies of this systematic review assessed the effects of observing non-referential gestures on information recall in TD children aged between 3 and 6 years. While three of these studies examined the effects on children’s recall of words [20,75,76], three others dealt with their ability to memorize spoken spatial directions or paths and event information [77,78,79], and the remaining one focused on their free recall of narratives [45]. Overall, 3 of the 7 experimental studies (two dealing with word recall and one with verbal spoken spatial directions) showed a positive effect of observing non-referential gestures on children’s information recall, whereas the remaining studies (one on word recall, two on verbal spoken paths and event information, and one on free narrative recall) did not.

Regarding the three studies that reported non-referential gestures having beneficial effects on recall, it is noteworthy that they presented the information in contexts that were pragmatically relevant for children. Two of them followed a within-subject experimental design [75,76], while the other one had a between-subjects design [77]. The study by [77] examined whether the presence of different gesture types in the verbal descriptions of a target path (presented in a single small-scale spatial array constructed from Lego materials and with a Lego character taking a certain route through the spatial array) would have an impact on the extent to which spatial information about this route (i.e., location and movement terms) was recalled by both adults and children. Participants were given the verbal description of the target path in the assigned condition and were then asked to recall the path (e.g., “Can you tell me the path that Lego man took through the scene without taking Lego man along with you?,” “Can you now show me the path that Lego man takes through the scene, taking Lego man along with you?”). A total of 95 adults (*M* = 28 years; *SD* = 7.6; range = 17 to 49) and 93 children (*M* = 4 years 3 months, *SD* = 4 months, range = 3 years 4 months to 4 years 9 months) participated in the experiment; however, the final sample consisted of 94 adults and 91 children. Participants were randomly assigned to one of three conditions: combined gesture (including 5 deictic, 5 beat, 5 iconic, and 5 metaphoric gestures), beat gesture, or no gesture. Results revealed that children in either the combined gesture condition or the beat gesture condition showed better verbal recall of spoken spatial directions than children in the no gesture condition. Overall statistical analysis using ANOVA found a large main effect of age group (*F*(1, 173) = 272.73, *p* < 0.001, partial η^2^ = 0.61), showing that the children recalled less information than the adults, and a small main effect of gesture condition (*F*(2, 173) = 3.17, *p* = 0.045, partial η^2^ = 0.03), revealing that gesture conditions (beat or combined) were more beneficial than the no gesture condition for information recall (ANOVA effect sizes are interpreted following the benchmarks suggested by [80].). The researchers took the average of the two gesture conditions and created a new “gesture” vs. “no gesture” variable and calculated the interaction between this new variable and age. A significant interaction between age group and the difference between the no gesture condition and the average of the two gesture conditions was found (*F*(1, 179) = 5.16, *p* = 0.024), with a small effect (partial η^2^ = 0.03), revealing that the difference in recall between age groups was greater for the no gesture condition than for the gesture conditions. Simple effects showed that for the total recall of spatial information by children there was a significant difference between the no gesture condition and the average of the two gesture conditions (*F*(1, 179) = 9.75, *p* = 0.002), with a small effect (partial η^2^ = 0.05). As for the adults, no significant differences were found between the no gesture condition and the average of the two gesture conditions on the total recall of spatial information (*F*(1, 179) = 0.006, *p* = 0.934, partial η^2^ < 0.01). Further, no significant difference between the two gesture conditions was found (*p* > 0.05) and there was no interaction between age and the two gesture conditions (*F*(1, 179) = 0.36, *p* = 0.551, partial η^2^ < 0.01).

In their within-subject study, [75] investigated whether the presence of non-referential gestures would improve word recall in 106 children aged 3 to 5 years (3-year-olds: *M* = 41.74 months, *SD* = 3.58; 4-year-olds: *M* = 53.93 months, *SD* = 3.79; 5-year-olds: *M* = 64.91 months, *SD* = 3.16) when presented with a list of things that Elmer, an absent-minded elephant, needed to remember before he went on a trip. Children completed the experimental task under two different audiovisual conditions, a beat condition and a no-beat condition, presented successively in counterbalanced orders. In the beat condition, each target word was accompanied by a beat gesture, whereas in the no-beat condition no beat gestures were used. Children were asked whether they could help Elmer to remember all the items on his to-do list, as he was very absent-minded and would really appreciate their help. Statistical analysis of the results using a Generalized Linear Mixed Model (GLMM) only showed a main effect of condition (*F*(1, 418) = 4.01, *p* < 0.05), indicating that the children recalled significantly more words in the beat condition than in the no-beat condition (β = 0.124, *SE* = 0.062, *p* < 0.05; no-beat condition: *M* = 0.38, *SD* = 0.48; beat condition: *M* = 0.49, *SD* = 0.50) (If the beta coefficient is positive, the interpretation is that for every 1-unit increase in the predictor variable, the outcome variable will increase by the beta coefficient value.). No effect of age (*F*(2, 418) = 2.80, *p* = 0.062) and no interaction between gesture condition and age (*F*(2, 418) = 0.11, *p* = 0.849) were found. Importantly, these findings show that observing non-referential gestures had a positive impact on word recall in children aged 3 to 5 years.

Along the same lines, the study by [76] showed that observing non-referential gestures can positively influence the memorization of contrastively focused items as well as information related to those items within contrastive discourse (i.e., containing a set of contrastively focused items). In this experiment, 51 4-year-old children (*M* = 4.57 years, *SD* = 0.26) were presented with discourse contexts in which a female human reminds Elmer the elephant about what they have done in their trips together (e.g., “Elmer, do you remember our trip to the field? In the morning, we went for a walk in the field. […] We noticed that near the lake there were roses and leaves, and you picked the roses […]”). These contexts contained a set of contrastively focused items (e.g., “roses and leaves”) in three conditions in a counterbalanced order: non-prominent speech, prominence in speech alone, and prominence in both speech and gesture (beat gestures). The children were then asked whether they could help Elmer to remember the questions that were related to what he and his friend had done together (e.g., “Now, help Elmer remember what he picked up when he went to the field. What did he pick up?”). A GLMM analysis of the results showed a significant main effect of condition (*F*(2, 288) = 5.28, *p* = 0.006), revealing that the children recalled more contrastively focused items in the prominence in both speech and gesture condition, compared to either of the speech-alone conditions (prominence in both speech and gesture vs. prominence only in speech, β = 0.241, *SE* = 0.077, *p* = 0.006; prominence in both speech and gesture vs. non-prominent speech, β = 0.191, *SE* = 0.081, *p* = 0.038). Moreover, regarding the proportion of items recalled between the prominence only in speech vs. non-prominent speech conditions, no difference was found (β = −0.050, *SE* = 0.082, *p* = 0.537).

By contrast, the 4 other studies found that observing non-referential gestures had no beneficial effect on children’s recall of information. In a study involving 30 adults (Experiment 1) and 36 4- to 5-year-old children (Experiment 2), [20] tested whether beat gestures and iconic gestures would enhance word recall by showing a video presentation of a list of verbs shown in isolation without a relevant discourse context in three counterbalanced within-subject experimental conditions: the verbs were accompanied by either an iconic gesture, a beat gesture or no gesture. Concerning the findings for the children, a main effect of gesture condition was found (*F*(2, 68) = 20.16, *p* < 0.001), with a large effect (η^2^ = 0.37), revealing that the children recalled a higher proportion of words in the iconic gesture condition than in either the beat gesture condition or the no gesture condition (*p* > 0.001). No significant difference between the beat gesture condition and the control condition was found, which indicates that non-referential gestures (i.e., the beat gesture condition) did not facilitate children’s word memory recall. However, results for the adults found a large main effect of condition (*F*(2, 56) = 7.87, *p* = 0.001, η^2^ = 0.21), showing that the participants displayed better recall scores when words were accompanied with iconic gestures than in the no gesture condition (*p* = 0.002), and when they were accompanied with beat gestures compared with the no gesture condition (*p* = 0.009). No difference between the number of items recalled in both gesture conditions was found. Further analyses comparing results from children and adults showed a large main effect of age group, revealing that the adults recalled a higher proportion of words than the children (*F*(1, 64) = 192.69, *p* < 0.001, η^2^ = 0.75), and a large main effect of condition (*F*(2, 128) = 22.11, *p* < 0.001, η^2^ = 0.26). A significant interaction between condition and group was found (*F*(2, 128) = 6.91, *p* = 0.001, η^2^ = 0.10). All in all, the proportion of words recalled was higher in the iconic gesture condition than in the no gesture condition for all participants. While the children recalled comparable proportions of words in the beat gesture and no gesture conditions, the adults recalled more words in the beat gesture condition than in the no gesture condition. These results suggest that non-referential gestures may entail a higher cognitive demand for children than for adults, who benefited from the presence of either iconic or beat gestures. In contrast to previous experimental designs, in this study each word was accompanied by a beat gesture, which may have reduced the highlighting function of beats. Moreover, the list of verbs was presented in isolation and not in a pragmatically natural discourse context.

Null results for non-referential gestures were also found in a study carried out by [78] in a between-subjects route direction task in which the participants were presented with a set of instructions to guide visitors through a zoo and then asked to recall and reconstruct the instructions. Like in [77], the goal was to examine the effects of gesture observation on the recall of the spatial information, but in this case the spatial direction task performed was larger in scale (i.e., when the spatial environment cannot be viewed from a single viewpoint). Participants were 172 3- to 5-year-old children (*M* = 4 years 5 months, *SD* = 4 months, range = 3 years 0 months to 5 years 4 months), who were randomly assigned to either the iconic/deictic gesture condition, the beat gesture condition, or the no gesture condition, and were presented with three videos of the head zoo-keeper verbally giving route directions through the zoo. In the two gesture conditions, key spatial or movements descriptors in the instructions were accompanied by nine gestures (e.g., “walk forward for a little bit,” “go past the frogs,” with underlined words indicating gesture points), which were either iconic/deictic or beat gestures, depending on the condition. A mixed-design ANOVA of the results revealed a main effect of gesture condition (*F*(2, 169) = 3.85, *p* = 0.023), with a small effect (partial η^2^ = 0.04), demonstrating that the children verbally recalled more items in the iconic/deictic gesture condition than in the beat gesture condition (*F*(1, 169) = 6.30, *p* = 0.013), with a small effect (partial η^2^ = 0.04). Moreover, no difference between the no gesture condition and the average of the two gesture conditions was found in terms of the number of verbally recalled items (*F*(1, 169) = 1.56, *p* = 0.213, partial η^2^ = 0.01). A possible explanation given by the authors for these findings is that referential gestures “may be processed more deeply due to their semantic value, leading to great recall without the presence of environmental cues” [78] (p. 10). To explain the lack of effects of non-referential gestures, the authors point out that “it is possible that the communication of spatial information accompanied by either no gestures or beat gestures may have seemed unusual or odd to preschoolers given that they would usually experience such messages accompanied by iconic and deictic gestures” (p. 11).

Interestingly, a second experiment was performed within this study involving the same route direction task but using a more pragmatically relevant instruction for the child. In this case, the child was asked to go to a location in the zoo where he/she remembered the zookeeper giving a particular instruction, and the experimenter recorded the path of movement on a paper map of the zoo. A mixed-design ANOVA regarding cued recall (i.e., the amount of route recalled verbally and during physical route retracing) showed a medium-sized main effect of condition (*F*(2, 169) = 5.72, *p* = 0.004, partial η^2^ = 0.07), indicating that the children presented with the materials in the two gesture conditions (beat condition or iconic/deictic condition) reported more at cued recall than the children presented with no gesture (*F*(1, 169) = 10.06, *p* = 0.002), with a medium effect size (partial η^2^ = 0.06). No difference between the amount recalled at cued recall in the iconic/deictic and beat gesture conditions was found (*F*(1, 169) = 1.60, *p* = 0.208, partial η^2^ = 0.01). Importantly, in this second experiment, although iconic gestures improved the children’s recall most, beat gestures also had some positive effect, suggesting that “the benefit of beat gestures may be apparent only when recall is cued by the environment” [78] (p. 10).

In the study by [79], a total of 67 4- to 6-year-old (54–73 months) children (*M* = 64.00 months, *SD* = 4.97) and 54 adults (*M* = 21.50 years, *SD* = 1.95) were asked to listen to a story about a character who followed different paths to find her friend’s house and then recount the information they had heard. The story included path descriptions (five alternative routes with various details) which were followed by event sequences with no spatial content (e.g., path: “she walked around the mountains;” event: “she saw a bank and took a rest on the bank;” again, underlined words indicate gesture points). In total, the story consisted of ten sentences each accompanied by one gesture (the underlined segment in the preceding examples). In a between-subjects design, participants were randomly assigned to one of these three conditions: iconic gesture condition, in which participants observed the stories with iconic gestures depicting the described path or action; beat gesture condition, in which speech was accompanied with rhythmic hand movements; or no gesture condition, in which the participant heard the narrative without any gesture. Participants were then asked a free recall-eliciting question (e.g., “Can you tell me everything you remember from the story?”) and their answers were subsequently scored for amount of content by a researcher. A mixed-design ANOVA found a large main effect of age group (*F*(1, 119) = 117.31, *p* < 0.01, partial η^2^ = 0.51), showing that the adults recalled more information than the children, and also a medium-sized main effect of gesture condition (*F*(2, 119) = 3.92, *p* = 0.022, partial η^2^ = 0.07). Post hoc analyses showed that when participants observed iconic gestures, they recalled more information than in the other conditions (beat gesture and no gesture condition) (Bonferroni, *ps* < 0.05). No significant interaction between age group and gesture condition was found (*F*(2, 119) = 0.39, *p* = 0.676, partial η^2^ = 0.007). Descriptive statistics showed that, for the children, the mean total free recall was 27.06 (*SD* = 12.30, min = 0, max = 45) in the iconic gesture condition, 21.63 (*SD* = 15.38, min = 0, max = 52.5) in the beat gesture condition, and 20.34 (*SD* = 12.82, min = 0, max = 45) in the no gesture condition. As for the adults, the mean total free recall was 60.29 (*SD* = 14.22, min = 40, max = 92.5) in the iconic gesture condition, 45.00 (*SD* = 22.64, min = 0, max = 95) in the beat gesture condition, and 48.44 (*SD* = 18.84, min = 15, max = 72.5) in the no gesture condition. It is important to mention that in this experiment, iconic gestures merely reinforced content and did not provide additional information. Therefore, these findings suggest that iconic gestures not only provide semantic cue, but also direct attention to certain parts of the story. By contrast, although the beat gestures were embedded in sentences to highlight target information in a naturalistic fashion, they made no contribution to participants’ recall performance.

Finally, the study by [45] reported no enhancement effects of beat gestures on the free narrative recall of 101 preschoolers aged 3.25 to 5.58 years (*M* = 4.65 years, *SD* = 0.47). In this experiment with a between-subjects design, children were asked to watch a video of a storyteller telling a two-minute narrative about a girl’s afternoon at the park with her family in one of four randomly assigned gesture conditions: iconic gesture, deictic gesture, beat gesture, or no gesture. In all conditions, gestures occurred at a total of ten points in the story. In the iconic gesture condition, the gestures represented the shape or action of the object described in the speech; in the deictic gesture condition, the gestures indicated the position of items referred to in the speech; and in the beat gesture condition the narrator produced rhythmic hand movements with no representational meaning and in focused positions. The different gesture types occurred at the same points in the narrative across conditions. After they had watched the video, the children were asked a free recall-eliciting question (e.g., “Please tell me everything you remember about the story you saw told on the computer”). Results demonstrated that children in the iconic and deictic gesture conditions scored higher on recall task than children in either the beat gesture or no gesture condition, between which there were no differences. A one-way between-groups ANOVA showed a main large effect of gesture condition on narrative recall (*F*(3, 97) = 6.69, *p* < 0.0005, partial η^2^ = 0.17). Further analyses showed that observing iconic gestures (*F*(1, 97) = 10.14, *p* = 0.010, partial η^2^ = 0.09, with a medium effect size) or deictic gestures (*F*(1, 97) = 18.17, *p* < 0.0005, partial η^2^ = 0.16, with a large effect size) increased narrative recall compared with no gesture condition. No other comparisons were found to be significant (*p* > 0.10). Perhaps unsurprisingly, when the recall of information available only through gestures and not present in the content of the narrative was analyzed, pairwise comparisons in a binary logistic regression (χ^2^(3) = 14.33, *p* = 0.002) found that the odds of reporting this information was higher for the deictic (B = 2.23, Wald = 10.59, *p* = 0.001, odds ratio = 9.33) and iconic (B = 1.74, Wald = 6.60, *p* = 0.01, odds ratio = 5.69) conditions than for the no gesture condition (The odds of success are defined as the ratio of the probability of success over the probability of failure. An odds ratio greater than 1 is a positive association (i.e., higher number of the predictor means group 1 in the outcome), while an odds ratio less than 1 is a negative association (i.e., higher number for the predictor means group 0 in the outcome). However, results for the beat gesture condition did not differ in this regard from those for the control condition (B = 1.02, Wald = 2.24, *p* = 0.14, odds ratio = 2.78), indicating that these gestures conferred no advantage.

All in all, it is worth noting that the three abovementioned studies reporting benefits of exposure to non-referential gestures relied on naturalistic uses of non-referential gestures in their experimental materials and assessed their role within discourse contexts that were pragmatically relevant for preschool and school children (e.g., small-scale route directions, list of things that an elephant needed to do before travelling, contrastive discourse).

#### 3.1.2. Narrative Discourse Comprehension

Two of 11 of the papers included in this systematic review addressed the potential role of observing non-referential gestures on narrative comprehension processes using a between-subjects experimental design. These two papers were also reviewed in the preceding section (see Section 3.1.1) because they analyzed the effects of observing non-referential gestures on information recall. As in that section, while the study by [76] showed positive effects of non-referential gestures in 5- and 6-year-old children’s narrative comprehension, the study by [45] found no such benefits (though the age of their participants was somewhat lower at 3.25–5.58 years).

The second part of the study by [76] tested the benefits of observing beat gestures in 55 5- and 6-year-old children (*M* = 5.84 years, *SD* = 0.56) in a narrative discourse task. Each participant was randomly assigned to one of two between-subjects conditions: beat gesture condition and no-beat gesture condition. In the no-beat condition, discourses were presented with prosodic prominence and no beat gestures in the target words, while in the beat condition, discourses were with prosodic prominence and with beat gestures in the target words (i.e., both performed on discourse markers and focal content words). The children were first shown a set of videos in which a storyteller told––with or without gestures accompanying discourse markers and focal content words––short six one-minute stories involving some farm animals that were friends of a sheep. After viewing each video, the children were asked to help the sheep find out what had happened to each animal and were asked two comprehension questions (e.g., “Why did the pig have to go home back early?” and “So how did the pig solve his problem?”). The children’s responses to the questions were then scored for comprehension. The results of a statistical GLMM analysis revealed a significant main effect of condition (*F*(1, 657) = 4.21, β = 0.572, *SE* = 0.279, *p* = 0.041, odds ratio = 1.772), indicating that the children comprehended the stories better when they were performed with beat gestures. It is important to note that in order to design the experimental materials, a preliminary study was conducted to determine what kinds of beat gestures naturally accompany child-directed narratives and at what points in the narratives they are typically used. This preliminary study guided both the form of the non-referential gestures and the placement of those gestures within the narrative discourse.

These results contrast with the findings of a second task reported in [45], in which (as described above) preschool children were asked to listen to a two-minute story in either iconic gesture, deictic gesture, beat gesture, or no gesture conditions. However, this second task was intended to test the effect of gesture conditions on the children’s narrative comprehension. Thus, in this task, after they had been exposed to the story, the children were asked 15 randomized specific questions related to the content of the narrative. As previously mentioned, the gestures only occurred at ten places in the narrative. Importantly, in the beat gesture condition, rhythmic hand movements without reflecting contextual meaning of the speech were performed in focused positions within discourse. Five of these questions took into account general story content (non-gesture-related questions), another five concerned gestures that reinforced but did not add to story content (redundant gesture-related questions), and the other five concerned gestures that conveyed information not present in the verbal narrative (non-redundant gesture-related questions). Results demonstrated that while the beat gesture condition and no gesture condition yielded similar narrative comprehension scores, meaning that beat gestures in no way enhanced comprehension, iconic and deictic gestures led to higher scores. Analyses to determine the effect of condition on non-gesture-related question scores using a one-way between-groups ANOVA found no significant difference between conditions in terms of narrative comprehension (*F*(3, 97) = 2.19, *p* = 0.093, partial η^2^ = 0.06). Finally, results on the effect of condition on gesture-related question scores using a one-way between-groups ANOVA showed a main large effect of gesture condition (*F*(3, 97) = 6.45, *p* < 0.0005, partial η^2^ = 0.17). Further analyses found the same outcomes as in the free recall results (iconic, *F*(1, 97) = 10.37, *p* = 0.009, partial η^2^ = 0.10, with a medium effect size; deictics, *F*(1, 97) = 6.98, *p* = 0.047, partial η^2^ = 0.07, with a medium effect size). Moreover, children produced higher comprehension scores on gesture-related items when they were accompanied by iconic (*F*(1, 97) = 12.34, *p* = 0.004, partial η^2^ = 0.11, with a medium effect size) or deictic (*F*(1, 97) = 8.58, *p* = 0.022, partial η^2^ = 0.08, with a medium effect size) gestures relative to children in the beat gesture and no gesture conditions, scores from which showed no significant differences (*p* = 0.994). Differences between scores in the iconic and deictic gesture conditions were likewise not significant (*p* = 0.938).

A potential reason for the difference between the results yielded respectively by [76] and [45] lies in the experimental materials employed. While (as noted above) the former study conducted a preliminary study in order to construct a more natural set of experimental materials, this was not the case in the latter study. In our view, it is important that beat gestures in discourse are assessed in terms of both the shape of the hand during the gesture and the point in the narrative at which the gesture occur, because both factors can mediate the gesture’s effect.

### 3.2. Predictive Effects

Two of the articles selected for this review were recent longitudinal studies that examined the predictive effects of the early frequency of use of non-referential beat gestures in children’s later more complex linguistic skills [68,81]. While both studies addressed predictive effects, they differed in two aspects. While the study by [68] examined the effects of 45 children’s production of non-referential gestures between 14 and 58 months of age in parent-child naturalistic interactions, the study by [81] tested the effects of the production of these gestures in older 5- to 6-year-old children while performing narrative discourses.

The main objective of the longitudinal study by [68] was to investigate whether the early production of non-referential beat and flip gestures (Non-referential flip gestures, a subtype of non-referential gestures, are performed by turning the wrist of the hand and opening it up to present the flat palm, accompanied or not with a shrug of the shoulders. They typically convey a judgmental or epistemic value of ignorance (e.g., [82])) (vs. referential iconic gestures) produced by 45 children in the total developmental window from 14 to 58 months of age predicted later narrative productions at 60 months (5 years old), measured in terms of narrative structure scores. On average, the children produced 1.19 beat gestures per session (*SD* = 1.74, range = 0 to 10.23), 1.86 flips per session (*SD* = 1.87, range = 0.15 to 9.15) and 3.58 iconic gestures per session (*SD* = 2.73, range = 0.31 to 11.46). Results from a GLMM analysis showed that the average number of beat gestures produced at baseline significantly predicted narrative skills at age 5 (β = 0.299, *SE* = 0.111, *z* = 2.689, *p* < 0.01). By contrast, the average number of flips (β = −0.163, *SE* = 0.109, *z* = −1.489, *p* = 0.137) and iconic gestures (β = 0.029, *SE* = 0.077, *z* = 0.381, *p* = 0.703) did not predict later narrative productions. This model explained 88.4% of the variance in children’s narrative outcomes (*R*^2^ = 0.884). Moreover, a second GLMM analysis also showed that the average number of non-referential beat gestures produced between 14 and 42 months of age were still predictors of children’s later narrative productions at age 5 (β = 1.386, *SE* = 0.583, *z* = 2.377, *p* = 0.017), while no significant effect was found for flips (β = −0.136, *SE* = 0.112, *z* = −1.212, *p* = 0.225) or iconic gestures (β = 0.009, *SE* = 0.067, *z* = 0.137, *p* = 0.891). This model explained 80.1% of the variance in children’s narrative outcomes (*R*^2^ = 0.801).

The second longitudinal study [81] reported the predictive value of both referential and non-referential gestures produced during narrative discourse by 5- to 6-year-olds (*M* = 5.9 years, *SD* = 0.55) on their later narrative productions (measured in terms of structural wellformedness) two years later, at 7 to 9 years of age (*M* = 7.98 years, *SD* = 0.60). On average, when they were 5–6 years the children produced 0.90 referential iconic gestures (*SD* = 1.54, *n* = 149) and 0.63 non-referential beat gestures (*SD* = 0.91, *n* = 105) in their narratives. A linear stepwise regression analysis was run to predict their narrative abilities at 7–9 years old based on the number of referential iconic gestures and non-referential beat gestures the children produced in their narratives at 5–6 years of age (*F*(1, 81) = 5.64, *p* = 0.020). Results demonstrated that the use of referential iconic gestures during narrative performance at 5–6 years predicted narrative structure scores two years later, when children were 7–9 years of age (β = 0.154, *SE* = 0.065, *p* = 0.020). However, no significant results were found for non-referential beat gestures (*p* = 0.432).

### 3.3. Causal Effects

Only two of the studies included in this review assessed the possible causal effects of narrative training that includes non-referential beat gestures in children’s narrative performance. Both studies involved 5- and 6-year-old children and used a between-subjects pretest-posttest experimental design. However, the studies differed in the main goal of the research. While the study by [83] examined the effects of having children observe beat gestures as part of a short narrative training task on their narrative performance in a posttest, the study by [84] investigated whether encouraging children to produce beat gestures could also affect their subsequent narrative performance.

In the first of these studies [83], following a pretest measuring their ability to produce a well-formed narrative, 44 5- and 6-year-old children (*M* = 5.94 years, *SD* = 0.57) underwent training which involved watching six one-minute stories presented under two randomly assigned experimental conditions: a beat gesture condition, in which a storyteller performed a narrative with prosodic prominence and beat gestures whenever she said a discourse marker or focal content word, and a no-beat gesture condition, where narratives were performed with prosodic prominence and no beat gestures in target positions within the story. Again, a preliminary study was carried out to identify the types of beat gestures that are spontaneously produced in child-directed narratives as well as to detect at what points in the narrative discourse these beat gestures tend to occur in natural circumstances. Children were simply asked to observe the stories. Children’s pretest and posttest narratives were then scored and compared by a researcher in terms of their structural wellformedness. Results of a GLMM analysis examining condition against structural wellformedness scores showed a main effect of condition (*F*(1, 172) = 8.04, *p* = 0.005), specifically in the beat gesture condition (β = 0.441, *SE* = 0.156, *p* = 0.005); and a main effect of test (*F*(1, 172) = 19.69, *p* < 0.001), with better posttest narrative structure scores than pretest scores (β = 0.597, *SE* = 0.135, *p* < 0.001). Moreover, the interaction between condition and test was found to be significant (*F*(1, 172) = 4.71, *p* = 0.031). Further post hoc analyses showed that gesture conditions differed in the posttest part, showing that higher narrative structure scores were produced by children in the beat gesture condition (β = 0.733, *SE* = 0.207, *p* < 0.001) than in the no-beat gesture condition. However, differences in gesture conditions were not reflected in pretest scores (β = 0.149, *SE* = 0.205, *p* = 0.467). Significant differences between pretest and posttest narrative scores were found in the beat gesture condition, with better scores in the posttest (β = 0.889, *SE* = 0.186, *p* < 0.001) than in the pretest. Differences between pretest and posttest scores in the no-beat gesture condition were not found to be significant (*p* = 0.119).

The second study [84] used the same narrative training paradigm employed in the previous study but assessed whether having children not only observe but also encouraging them to produce beat gestures would enhance the effects seen in [83]. In this case, 47 5- to 6-year-old children (*M* = 5.92 years, *SD* = 0.54) were randomly assigned to one of two experimental conditions: beat encouraging condition and beat non-encouraging condition. Following a pretest which measured not only structural wellformedness but also fluency on their narrative output, the children were shown videos of the same six narratives used in the previous study, though in this case both groups saw the version of the video in which the storyteller performed prosodic prominence and beat gestures in target positions. Children were then asked to retell the story they had just heard. However, while children in the beat non-encouraging condition were asked to retell the stories without any instructions regarding gesture, in the beat encouraging condition they were encouraged to use hand movements (i.e., beat gestures) like those they had seen the storyteller use while recounting what they had heard. Children’s pretest and posttest narratives were then scored and compared. Results from a first GLMM analysis found a main effect of test (*F*(1, 184) = 25.19, *p* < 0.001), with higher narrative structure scores in the posttest (β = 0.834, *SE* = 0.166, *p* < 0.001) than in the pretest, and a significant interaction between condition and test (*F*(1, 184) = 6.17, *p* = 0.014). Further post hoc analyses revealed that the gesture conditions differed in posttest narrative structure scores, with higher narrative structure scores in the beat encouraging condition (β = 0.697, *SE* = 0.265, *p* = 0.009) than in the beat non-encouraging condition. However, conditions did not differ in terms of pretest scores (β = 0.129, *SE* = 0.265, *p* = 0.628). Significant differences between pretest and posttest narrative scores were found in the beat encouraging condition, with higher scores in the posttest (β = 1.246, *SE* = 0.240, *p* < 0.001) than in the pretest. Differences between pretest and posttest scores in the beat non-encouraging condition were not found to be significant (*p* = 0.069). A second GLMM analysis revealed a main effect of test (*F*(1, 184) = 18.28, *p* < 0.001), with higher fluency scores in the posttest (β = 0.803, *SE* = 0.188, *p* < 0.001) than in the pretest, and an interaction between condition and test (*F*(1, 184) = 4.65, *p* = 0.032). Further post hoc analyses showed no significant difference between pretest scores (β = 0.214, *SE* = 0.468, *p* = 0.647) and also posttest scores (β = 0.596, *SE* = 0.533, *p* = 0.265) across conditions. Moreover, pretest and posttest scores for the beat non-encouraging condition did not significantly differ (β = 0.398, *SE* = 0.249, *p* = 0.112). However, pretest and posttest scores did differ for the beat encouraging condition, with higher fluency scores in the posttest (β = 1.208, *SE* = 0.281, *p* < 0.001) than in the pretest.

Overall, the two studies showed that either asking children to observe, or encouraging them to produce non-referential gestures in a short narrative training task, had immediate short-term effects on their narrative performance in terms of both narrative structure and narrative fluency.

## 4. Discussion and Conclusions

The aim of this systematic review was to search for and compare the findings of any experimental research that addressed the question of whether non-referential gestures can play a scaffolding role in both children’s cognitive and linguistic abilities, as well as in the development of more complex language skills, like narrative performance. A total of 11 articles, all published within the last decade, met the eligibility requirements for inclusion. These studies ––some within-subject and others between–subjects in design––measured the effect of non-referential gestures on three different domains of cognitive or linguistic skill, namely information recall, narrative discourse comprehension, and oral narrative discourse performance. Immediate comparison of study findings was therefore only possible when the studies explored the same domains. At the same time, their findings revealed the presence or absence of three sorts of effects, namely association effects, predictive effects, or causal effects, leading to our three fundamental research questions. Importantly, it should be noted that there is a discrepancy in the number of studies concerning the different outcome measures. While seven papers are reporting recall and comprehension effects, only two articles focus on causal effects, and two more on predictive effects. In what follows, we will discuss what light this collective body of research sheds on each of these areas.

It must first be noted that the results of these 11 studies are not in full agreement. With regard to the effect of observing non-referential gestures on information recall, the contradictory findings can be explained by two factors, namely the pragmatic appropriateness and complexity of the task for child participants on the one hand; and on the other the choice of stimuli/materials used in each study. First of all, the studies that reported positive results [75,76,77] used ecologically valid instances of non-referential gestures in tasks that were pragmatically appropriate for children (small-scale route directions in [77]; a list of things that an elephant needs to do before travelling in [75]; contrastive discourse in [76]). On the other hand, although both studies by Austin and Sweller used pragmatically appropriate contexts, it may be that the larger scale route directions that the children had to recall in [78] nullified the potential benefit of non-referential gestures, which was not the case for the less complex and small-scale spatial array employed in [77]. The null results in [20] and [79] could also be explained by the lack of pragmatic appropriateness in the task for 4- to 6-year-olds. While [20] presented the gesture stimuli in isolation (i.e., lists of verbs accompanied by iconic gestures, beat gestures, or no gestures) and not in a pragmatically felicitous discourse context, [79] asked children to remember a list of sentences of a story that included both path descriptions and event sequences. Moreover, in relation to the naturalness of the experimental materials, and specifically the appropriateness of gesture co-occurrence with specific target words, the study by [79] and another study with null results [78] used beat gestures in co-occurrence with both path and event information (e.g., with target prepositions encoding spatial information, like “walk forward for a little bit”, with underlined word indicating gesture point), which the authors themselves acknowledged might be perceived as unnatural. For instance, [78] note that “the communication of spatial information accompanied by either no gestures or beat gestures may have seemed unusual or odd to preschoolers given that they would usually experience such messages accompanied by iconic and deictic gestures” (p. 10). Therefore, it could be that beat gestures co-occurring with these target words did not seem natural to the participating children.

Regarding the influence of non-referential gestures in narrative comprehension processes, the contradictory results might again be related to the stimuli used. First, as we have noted, in order to ensure the validity and naturalness of the experimental materials, the study by [76] conducted a preliminary study prior to the experiment in order to determine precisely what kinds of non-referential gestures naturally accompany child-directed narratives and at what points they typically occur within the discourse. On the basis of this preliminary study, beat gestures were used in the experiment to highlight both focal content words and discourse markers. In [45], by contrast, gestures were simply placed at ten places in the narrative. Moreover, another issue to be considered is the number of gestures relative to the length of the narrative: while the stories in [76] were relatively short narratives containing between eight and eleven beat gestures each, the stories in [45] were four times longer and contained ten gestures each.

The two longitudinal studies that aimed to address the predictive role of non-referential gestures also yielded contradictory results. On the one hand, [68] provided evidence that the early frequency of use of non-referential beat gestures produced during naturalistic parent-child interactions in the developmental window from 14 to 58 months was predictive of higher narrative skill levels later at 60 months. These results contrasted with the lack of predictive value offered by non-referential flip gestures and referential iconic gestures. On the other hand, [81] examined the predictive value of both referential iconic gestures and non-referential beat gestures produced in narrative discourses by children aged 5–6 for the quality of their narrative production at 7–9 years. In this case, results did not show non-referential gestures having significant predictive value. These null results may be due to the higher number of referential iconic gestures produced at 5–6 years of age, which might have been triggered by the narrative retelling task. Another explanation for the different predictive results between studies could be related to the fact that in naturalistic parent-child interactions, children might have included all kinds of referential iconic gesture types, whereas referential iconics produced in narrative corpora could also include different viewpoints in narrative (e.g., CVPT or “observer-viewpoint”, OVPT, gestures [6], in line with [47]). All these factors might have reduced the effect of non-referential gestures, whose use has been demonstrated to significantly increase with age in narrative development [58]. All in all, further studies should investigate the predictive effects of the use of non-referential gestures for later stages of narrative production, when such gestures occur more frequently and are thus more stably acquired in complex narrative discourses.

Finally, the two training studies in our selection revealed that training in oral narratives using non-referential gestures offers benefits, in terms of not only narrative structure but also oral fluency [83,84]. Both studies showed that a brief training session with non-referential gestures is valuable for narrative production, revealing a causal link between these gestures and narrative gains in the production of narratives ––a complex linguistic skill––at 5 to 6 years of age.

Overall, though the findings reviewed in this manuscript are mixed regarding the effects of observing non-referential gestures for recall and comprehension, results were positive when these were used in pragmatically relevant and non-complex tasks for children and when they reflected a natural co-occurrence with target words. Six of the 11 studies assessed in this systematic review provide evidence of the positive effects of non-referential gestures in children’s information recall, narrative discourse comprehension processes, and oral narrative discourse performance. Even though the empirical evidence of the value of non-referential gestures is not yet as strong as the evidence in favor of referential gestures, it is clear that there are sufficient grounds to claim that non-referential gestures play an important role in boosting children’s learning and language development. It is of interest to note that the mixed findings obtained in the developmental literature resemble the contradictory patterns reported by studies assessing the role of non-referential gestures in adult speech processing (see [85] for a review). While some research has shown that non-referential gestures positively affect adults’ ability to recall information [20,86,87], this has not been true of other studies [77,79,88] (see also [85]). According to [85], a potential reason for the lack of the beneficial effect of non-referential gestures reported in some studies could be related to the stimuli used, as positive results have generally been shown when non-referential gestures are used in pragmatically natural and restricted contexts, such as for marking contrastively focused information. Similar to the child experiments reviewed here, it is clear that experiments involving non-referential gestures that use ecologically valid tasks and materials have reported positive results.

In general, the present systematic review points to the need for further research to assess the role of non-referential gestures. In terms of methodology, experiment design must clearly take into account task appropriateness as well as the pragmatic function of non-referential gestures in discourse. This is because non-referential gestures emphasizing some spatial or event information can be perceived as unnatural in discourse, and thus it is important to assess which parts of the discourse the speaker should accompany with non-referential gestures. Conducting previous preliminary analyses can help to precisely define the visual features of gestures as well as their patterns of association with target words in a natural and spontaneous context (see the preliminary study used by [83]), thereby ensuring the ecological validity of the experimental materials.

All things considered, the evidence presented here would seem to support the view that the significant bootstrapping and predictive role of non-referential gestures is related to the pragmatic, discursive, and prosodic functions they perform in discourse. Non-referential gestures may serve important linguistic functions in discourse, associated with rhythmic marking, discourse structure marking, and information structure marking ([6,48,50,51,62,63,67], and others), such as new or accessible referents in discourse [62,63]. The developmental findings reported in the present review also lend support to the hypothesis that non-referential gestures develop in parallel with narrative development [54,57] (see also [58]). Importantly, non-referential gestures can help children focus on critical parts of a story, by providing them with visual markers that facilitate the parsing and processing of narrative discourse.

Though the studies here represent a first step in this direction, further research is needed to evaluate the potential of narrative training and sociopragmatic paradigms that include a strong multimodal component involving both referential and non-referential gestures. The fact that non-referential gestures are a strong discourse framing mechanism ([48,51,67], and others) is an indication that they might constitute a powerful tool for assessment in TD populations. We reviewed here two studies in which non-referential gestures were successfully used in narratives to improve the narrative production skills of TD children [83,84]. Moreover, future investigations could extend these findings to populations with language disorders. In this sense, the inclusion of non-referential gestures could be of benefit in language intervention programs for non-TD children. Reinforcing the production of these types of gestures during narratives might provide children with an important means of non-verbal discourse marking that can help them improve their narrative and interactional abilities.

Interestingly, some recent studies assessing multimodal training have suggested that such techniques can enhance children’s social cognition and expressive pragmatic skills [89] and that having children observe audio-visual stimuli involving all kinds of gestures can improve their narrative productions [90]. On the one hand, the study by [89] showed that 3- to 4-year-old preschoolers improved their expressive pragmatic skill through training in which they were asked to embody mental states using prosodic and gestural cues. On the other hand, findings in [90] revealed that both 5-year-old children with early brain injury (who had difficulty in structuring narrative) and TD children were more likely to produce better-structured narrative retelling when the storyteller performed story-relevant gestures while speaking. All in all, additional research exploring the multimodal components of narrative and sociopragmatic treatments is needed. Our view is that the spontaneous use of gesture in discourse involves a combination of referential and non-referential gestures, and that non-referential gestures cannot be neglected as they have an important function in multimodal trainings.

Concerning the findings of the reported quantitative studies, we consider that future research could address the application of gesture-based narrative interventions under more specific populations, considering for example non-TD children’s language and communication, which could be of help for clinicians, teachers, families, and researchers concerned with language development in such children. Various classroom training studies involving narratives have already been successfully carried out with preschool non-TD children (e.g., [91,92]). In this regard, [92] demonstrated that narrative interventions are a promising and effective strategy to teach oral narration to children with risk factors and narrative language delays, who may benefit from it in terms of their short-term and long-term narrative retelling skills (see also [93,94,95,96]). We claim that more effective interventions should include training focused on not only children’s speech but also their gestures and general multimodal behavior. An example of this is the study by [91], which proposed an intervention based on activities combining voluntary storytelling with group story-acting carried out as a regular part of the preschool curriculum (see also [97], for the benefits in social competence of theatre-based intervention involving role-playing, improvisation, and play performance with autistic children). The results showed that story-acting training (i.e., story dictation and dramatization) promoted the abilities of preschool children from low-income and otherwise disadvantaged backgrounds in three major areas that contribute to their readiness for success in formal education, namely narrative and other oral language skills, emergent literacy, and social competence.

Previous systematic review and meta-analysis studies [98,99,100] have provided evidence that using social pragmatic, pragmatic language, and narrative interventions can support the social communication and language abilities of children with ASD or with other language disorders. However, to our knowledge, there are no studies that have assessed whether multimodal (gesture-based) training with non-TD children could also contribute to their language development. We claim that paying attention to the gestures that learners produce can help professionals determine any existing underlying delays in acquiring more complex linguistic or cognitive skills in populations with atypical development. As gestures are likely to give clues not yet evident in their speech about a learner’s understanding of a task, this can help professionals determine whether learners are ready to take further steps in their learning. Also, gestures can help diagnose any existing language or cognitive difficulties (see [101,102,103,104,105] for reviews) that result in an atypical language profile (e.g., children with early brain injury, autism, Down syndrome, etc.; [106,107]). Because both narrative production and gesture can index individual differences in typical development profiles, a better understanding of gesture-speech development could help improve clinical practices regarding children’s language assessment and intervention. All things considered, these reviewed studies may extend the findings of the TD children to non-TD children, by offering clinicians and speech-language therapists some guidance by highlighting the importance of including gestures in their cognitive and linguistic assessment tasks.

Although there are two training studies with TD children that have been reviewed in this paper [83,84], training studies conducted with non-TD children have to date not focused specifically on the role of multimodality. For instance, no previous training studies involving gestures as an empirical condition have assessed the value of narrative and sociopragmatic training in ASD or in language disorders [98,99,100]. The long-term effects of these interventions and the extent to which learning thus acquired is generalized to new contexts is largely unknown, and thus assessing multimodal interventions could help teachers, clinicians, speech-language therapists, and also families to adapt new teaching methodologies that emphasize the importance of the role of gestures in multimodal narrative abilities in children. This suggests that the present study would be aptly complemented by a systematic review covering research on multimodal interventions/training in both TD and non-TD populations.

In conclusion, the present systematic review should clarify the state of the art with regard to the link between non-referential gestures and children’s language development. Based on this review, we feel that it is safe to claim that non-referential gestures are likely to be helpful in both children’s cognitive development and their acquisition of complex linguistic skills, although further investigation is needed to confirm this conclusion. This impact could be deemed in both TD and extended to non-TD populations, as non-referential gestures can represent an important multimodal tool that can be used to build up and frame children’s processing and production of complex language.

## Figures and Tables

**Table 1:** Empirical studies included in the systematic review.

Author, Year	Study Population	Study Design	Control and Experimental Conditions	Outcome Measure	Did Non-Referential Gestures Have a Positive Effect on Children’s Outcome Measure?
Austin & Sweller (2014)	91 children (49 girls and 44 boys); *M* = 4 years 3 months, *SD* = 4 months; range = 3 years 4 months to 4 years 9 months; Australian-English speakers	Between-subjects experiment	(1) No gesture; (2) Beat gesture; (3) Combined gesture (5 beats, 5 deictics, 5 metaphorics, and 5 iconics)	Spatial information recall	Yes → Beat gesture condition and Combined gesture condition (vs. No gesture condition)
Austin & Sweller (2017)	172 children (original sample: 77 girls and 97 boys); *M* = 4 years 5 months, *SD* = 4 months, range = 3 years 0 months to 5 years 4 months; Australian-English speakers	Between-subjects experiment	(1) Iconic/deictic gesture; (2) Beat gesture; (3) No gesture	Spatial information recall and cued recall	No → Iconic/deictic gesture condition (vs. Beat gesture condition and No gesture condition)
Igualada et al. (2017)	106 children (47 girls, 59 boys); 3 years: *M* = 41.74, *SD* = 3.58; 4 years, *M* = 53.93, *SD* = 3.79; 5 years: *M* = 64.91, *SD* = 3.16; Catalan speakers	Within-subject experiment	(1) Beat; (2) No-beat	Word recall	Yes → Beat condition (vs. No-beat condition)
Kartalkanat & Göksun (2020)	67 children (original sample: 36 girls and 35 boys); *M* = 64.00 months, *SD* = 4.97; Turkish speakers	Between-subjects experiment	(1) Iconic gesture; (2) Beat gesture; (3) No gesture	Path and event information recall	No → Iconic gesture condition (vs. Beat gesture condition and No gesture condition)
Llanes-Coromina et al. (2018)	51 preschool children; *M* = 4.57, *SD* = 0.26; Catalan-Spanish bilingual speakers (Experiment 1)	Within-subject experiment (Experiment 1)	(1) Non-prominent speech; (2) Prominence in speech alone; (3) Prominence in both speech and gesture (beat gestures) (Experiment 1)	Information recall in contrastive discourse (Experiment 1)	Yes → Prominence in both speech and gesture condition (vs. Non-prominent speech and Prominence in speech alone conditions)
	55 children; *M* = 5.84, *SD* = 0.56; Catalan-Spanish bilingual speakers (Experiment 2)	Between-subjects experiment (Experiment 2)	(1) Beat; (2) No-beat (Experiment 2)	Narrative comprehension (Experiment 2)	Yes → Beat condition (vs. No-beat condition)
Macoun & Sweller (2016)	101 children (57 girls and 44 boys); girls: *M* = 4.62, *SD* = 0.40; boys: *M* = 4.70, *SD* = 0.57; total *M* = 4.65, *SD* = 0.47; Australian-English speakers	Between-subjects experiment	(1) Iconic gesture; (2) Deictic gesture; (3) Beat gesture; (4) No gesture	Narrative recall and narrative comprehension	No → Iconic gesture condition and Deictic gesture condition (vs. Beat gesture condition and No gesture condition)
So et al. (2012)	36 children (18 girls and 18 boys); 4 to 5 years; English speakers	Within-subject experiment	(1) Iconic gesture; (2) Beat gesture; (3) No gesture	Word recall	No → Iconic gesture condition (vs. Beat gesture condition and No gesture condition)
Vilà-Giménez et al. (2020)	83 children (43 girls, 40 boys); Time 1: *M* = 5.9, *SD* = 0.55; Time 2: *M* = 7.98, *SD* = 0.60; Catalan-Spanish bilingual speakers	Longitudinal	(1) Non-referential beat gesture; (2) Referential iconic gesture	Later oral narrative productions (narrative structure scores)	No → Referential iconic gestures (vs. Non-referential beat gestures)
Vilà-Giménez et al. (under review)	45 children; Time 1: between 14 to 58 months of age; Time 2: *M* = 6, *SD* = 0.42; American-English monolingual speakers	Longitudinal	(1) Non-referential beat gesture; (2) Non-referential flip gesture; (3) Referential iconic gesture	Later oral narrative productions (narrative structure scores)	Yes → Non-referential beat gestures (vs. non-referential flips and referential iconics)
Vilà-Giménez et al. (2019)	44 children (20 girls and 24 boys); *M* = 5.94 years; *SD* = 0.57; Catalan-Spanish bilingual speakers	Between-subjects training (pretest-posttest design)	(1) Beat; (2) No-beat	Oral narrative performance (narrative structure scores)	Yes → Beat condition (vs. No-beat condition)
Vilà-Giménez & Prieto (2020)	47 children; *M* = 5.92, *SD* = 0.54; Catalan-Spanish bilingual speakers	Between-subjects training study (pretest-posttest design)	(1) Beat encouraging; (2) Beat non-encouraging	Oral narrative performance (narrative structure and fluency scores)	Yes → Beat encouraging condition (vs. Beat non-encouraging condition)

## Data Availability

Not applicable.
